# Notes and Comments

**Published:** 1923-07

**Authors:** 


### NOTES AND COMMENTS.

### THE SHEFFIELD CONFERENCE.

IT is highly significant of the present situation of the Voluntary Hospitals that every one of the papers on the programme at the Conference of the British Hospitals Association at Sheffield, dealt more or less immediately with finance. Those of Sir Napier Burnett and Mr. H. J. Waring, which were conce ned mainly with paying patients, we have discussed in our first leading article. That of Lord Hambleden on the important subject of the pecuniary nexus between the hospitals and the Approved Societies will be of poignant interest to every Board of Governors, since it raises the very serious question of the permanency of the existing arrangement. Approved societies representing little more than half the insured persons in this country are co-operating, and there is no certainty of their continuance. It is of the first importance that the relations between the hospitals and the societies should be based on secure foundations, and Lord Hambleden's paper should be of substantial value in calling public attention to the instability of the existing arrangement. The Conference decisively rejected the proposal for the creation of a Provincial Central Fund on he lines of King Edward's Fund, chiefly, it would seem, from fear of centralisation and interference. Conditions in the provinces differ no doubt in many respects from those in the capital, but, as we said a year ago in commenting upon the discussion of the subject at the Liverpool Conference, such a fund is practically certain to be found necessary, sooner or later. The matter is one rather of the appropriate time than of abstract desirability.

### THE NEW TREATMENT FOR TUBERCULOSIS.

THERE have been so many disappointments about treatments for tuberculosis that it would be unwise to assume at once that the new method of dealing with it described by Dr. Georges Dreyer in his recent lecture at St. Mary's Hospital has solved the difficulty which has so long baffled the pathologist. But Dr. Dreyer, the distinguished Dane who is Professor of Pathology in the University of Oxford, has worked so cautiously and has obtained results so encouraging that the further progress of his experiments will be watched with the keenest interest and sympathy. Put into a sentence, his treatment consists in depriving the germs of tuberculosis of the fatty protection which saves them from the assaults of vaccines launched against them. From the defatted tubercle bacillus he has prepared an "antigen" which has given remarkable results. Having first succeeded in healing experimentally produced tuberculosis in guinea-pigs, he went on, with extreme scientific caution, to make tests with human cases, and experiments have been, and are still being, made at Brompton, at the London, and at Hampstead. At Brompton a "hopeless" case improved amazingly in a month; in anothe case, this time of tuberculous glands of the neck, with an ulcerated sinus, the first dose produced an improvement. So hopeful, indeed, is the treatment that the London Hospital has set apart a small ward for further investigations. Prolonged experiment will, no doubt, be necessary before it will be safe to assume that the victory has been won, but, at the worst, we can hardly doubt that a long step forward has been taken on the weary and thorny road towards ridding the world of one of its most deadly and intractable scourges.

### PRINCESS CHRISTIAN.

"FLORENCE NIGHTINGALE planted, Princese Christian watered, and God has given an increase in a very wonderful way." So spoke Mr. Bridgeman, the Home Secretary, when moving in the House of Commons a vote of condolence with the King upon the death of his aunt, Princess Christian. The words will find an echo wherever the great work which this devoted Royal lady accomplished for the profession of nursing and the welfare of hospitals is known. From her very girlhood Princess Christian, who ardently desired herself to be a nurse, never slackened in her activities in behalf of the sick and suffering—activities which were by no means confined to the establishing of her own Nursing Institute at Windsor and her Holiday Home for Crippled Boys. No member of the Royal Family suffered more grievously from war than she. The final campaign against the Boers cost her one son, and when the Great War came her other son's change of nationality cost her, in a very distressing sense, the loss of the other. Yet in each of these conflicts she organised a hospital train which did splendid work. Her life is an outstanding example of what a woman of high position, meagrely endowed with pecuniary means, can accomplish for the good of her fellows. Never was there a more conclusive proof of the old truth that personal service counts for infinitely moie than the mere giving of money, laudable as that will ever be.

### UNEMPLOYED AND UNWASHED.

IT is not at first sight obvious why men and women who are unhappily out of work should, with their children, be less cleanly than when they are employed. But Dr. Chetwood, the School Medical Officer of Sheffield, points out that the explanation is not psychological, as might have been supposed, but economic. His experience, and that of many others, suggests that in periods of prolonged trade depression children are sent to school not only poorly clothed, but dirty, because hot water involves the use of coal, and the first charge upon the small quantity of fuel available is for cooking. It is therefore the more unfortunate that, as a measure of economy, three out of the four school baths in Sheffield have been closed. When domestic bathing facilities are curtailed is surely the wrong time for seeking to save a little money by reducing communal facilities also. Cleanliness is not only next to godliness, but next to health, and, broadly speaking, the more water used for household and personal purposes, the more likely the community is to be healthy. Meanwhile it is melancholy indeed to reflect that to the unhappiness of unemployment to those who are willing and anxious to work should be added personal discomforts and privations which are positive dangers to the community.

### THE KING'S FUND.

PERHAPS the chief point brought forward at the annual meeting of King Edward's Hospital Fund for London was the honorary treasurer's statement that the position of the fund as regards investments was stronger than before the emergency distribution of £250,000 three years ago. This means, of course, that the consequent increase of income will occur now that the combined appeal is over. The ordinary income of the fund last year was £229,121, apart from the first instalment of the proceeds of the Combined Appeal. The sum distributed from the latter was £413,214. It was agreed that daily reports of vacant beds available at hospitals should be supplied to the police and ambulance authorities. The Prince of Wales's speech naturally dwelt upon the Combined Appeal and the causes which contributed to its result. The lesson he drew was that voluntary co-operation, under the leadership of the King's Fund, had proved its value. Omitting the income from special distributions, the Prince remarked that the net aggregate deficit of the hospitals for the year 1922 had been reduced to £171,000. It was £380,000 in 1920, and £210,000 in 1921. To bridge this gap is the problem of the future, to which the fund, as the Voluntary Hospitals Committee for London, is now addressing itself. There is no doubt that the fund has proved its usefulness no less during the emergency that followed the war than it had done before. It still continues to attract munificent legacies, and its position to-day, with a larger capital than it had before it realised a quarter of a million of its assets to meet the crisis of 1920, is the most convincing proof of its usefulness. The fund, indeed, is the most stable element in hospital finance. It maintains their revenue. It provides them, on emergency, with large capital sums. It has accomplished even more than its founder himself hoped.

### A CONCERTED CANCER CAMPAIGN.

IN 1921, the last year for which the Registrar-General's figures are available, there were in England and Wales 45,328 deaths from cancer of persons over thirty years of age. Within that limit of age one death in every seven was caused by the mysterious and murderous disease for which science has vainly sought a remedy. We know little of its cause and less of how to cure it ; few diseases have produced a larger or more varied crop of theories or, we fear it must be added, more virulent animosities within the realm of medicine. But, sooner or later, the right theory will be broached, and in the hope that concerted effort will achieve more than isolated experiment, the British Empire Cancer Campaign has been set on foot, and is asking for money with which to do the work it is undertaking. There will be working Committees representing every branch of the curative art, with an executive forming a central clearing house. The organisation and machinery of the British Red Cross Society have been placed at the disposal of the Council of the Campaign, and every help will be given to the work that is already being carried on. Co-ordination of effort and concerted action are certain to produce results of real value to humanity, even if they do not at present solve the enormous problem it is sought to master. The investigation must needs make a powerful appeal to the whole world, and we trust that sufficient money (which may be sent to any branch of Lloyd's Bank) will be forthcoming to lend it to an issue which will remove one of the most dreaded and widespread terrors that menace mankind.

### THE APOTHEOSIS OF THE TOMATO.

THE hygienist will tell us many things concerning the great value of fresh or green vegetable foods, but curiously enough very little is heard of the properties of tomatoes. Obviously this neglect is not justified. A recently published analysis from America proves that tomatoes contain quantities of all three recognised vitamines and this in higher proportion than any other fruit or vegetable. They carry, too, large amounts of citric, malic and phosphoric acids, which have been so long and so frequently used as remedial agents that they call for no detailed comment. Tomatoes taken as food do not give a large, useless residue ; they are relatively quickly and easily digested. Also contrary to the general view, the tinned products, or rather their juices, have, compared with other foods, extremely little action on the metal of the container.

### OCULIST AND OPTICIAN.

LAST month we printed a letter from a correspondent protesting against the usurpation by opticians of the difficult and delicate functions of the trained oculist. There are, we know, opticians who have obtained qualifications from their own technical organisations, and we are ready to admit that they are infinitely less dangerous than those—and they are the great majority—who do not possess the eye-testing diploma of the British Optical Association, or the Spectacle Makers' Company. But the treatment of the eye is an exceedingly impor ant department of science, requiring prolonged and specialised study and experience, and we are glad to see that the "Westminster Gazette" has carried the matter further than was possible in the short letter we printed in our June number. Everyone who is not an optician must surely agree with Dr. Cox, the Medical Secretary of the British Medical Association, that eye-testing should be confined only to "those who have gone through a medical examination." Great numbers of people suffer from defective eyesight, and only too often the trouble is caused by actual disease. It is unlikely, to say the least, that those who have not acquired the essential medical knowledge should be able to detect the presence of disease—they are, in a very real sense, the blind treating the blind. The subject is so important that we hope it will not be allowed to rest where it is. Modern life is not favourable to the preservation of good eyesight, and every year it becomes of greater moment that this department of science should be reserved to those who are really qualified to deal with it. The position would be less serious if we could be sure that every optician would send cases which present difficulty to an ophthalmic surgeon ; but in far too many cases he fails to do so.

### THE HIGHBURY HOSPITAL FIRE.

FIRES in hospitals are, happily, much more rare in this country than in America, where, as we recently recorded, the average is two a week. English hospitals are usually very solid constructions, able to oppose serious resistance to fire. When they are not the risk is great indeed, as the Highbury catastrophe makes sadly obvious. There is at Highbury, in the outskirts of Birmingham, familiar as the residence of the late Mr. Joseph Chamberlain, a Ministry of Pensions Hospital, not owned or administered by the Ministry, but allocated to its exclusive use. In broad daylight a fire broke out in an isolated open-air ward containing fifteen patients, only three of whom could help themselves. In less than five minutes the place was a roaring furnace, and, despite the efforts of the entire staff of the hospital, and the patients who were well enough to help, two patients lost their lives and two others were seriously injured, to say nothing of minor hurts to other patients and helpers. This fatal ward was of wood, on a brick foundation, with a galvanised iron roof, and " of the most modern type, such as is extensively used in this and other countries " for the treatment of tuberculosis and other diseases needing ample sun and air, and there were two exits, one of them wide enough to allow two beds to be removed through it simultaneously ; the other was instantly cut off by the flames. Nothing is definitely known as to the cause of the fire, but a searching inquiry is being made, not only into that, but into the suitability of this type of building for its purpose. This shocking disaster should be sufficient proof in itself that wooden huts are not suitable habitations for maimed and helpless people. We have not yet discovered how to make any kind of building really " fireproof," and it would seem to be beyond the wit of man to make wooden ones safe. We hope that the sad lesson of Highbury will be taken to heart at once, and that risks of this kind will be reduced with all possible rapidity.

### INSULIN IN ACTUAL USE.

SINCE we last recorded the progress made with the new diabetic treatment, matters have moved apace. Supplies have for a short time been available for trial in selec ed hospital cases, and a summary of the reports (6s.) has already been published by, he Medical Research Council. More recently Insulin has been successfully manufactured and actually placed on the market at the disposal of medical men, the main, and at the moment all important, condition being that the doctor should first make arrangements for correct laboratory control of the progress of his case. The essential observation required is periodic estimation of the amount of sugar present in the patient's circulating blood. In a normal person the blood-sugar, as it is called, remains at almost a constant level. In a sufferer from severe diabetes it is generally very high. The object of the new treatment is to reduce the amount of sugar in the blood, and if possible keep it at the normal level or nearly so. Therefore progress of a case cannot be correctly watched unless the blood analyses are made as the series of Insulin injections proceeds. There is, however, another reason for this laboratory control. As already explained, our normal circulating blood always contains a certain amount of sugar, and cannot correctly carry out its nutritional functions unless this level is maintained. Therefore, although in a diabetic person with a high sugar content in his blood we give Insulin to reduce that amount, we must be very careful not to reduce it too far, as more harm than good is done. The effect may even be dangerous. At the moment it is, at any rate, absolutely proved that Insulin injections will bring down and keep down the excess of sugar in the blood. No more sugar is passed and the patient, if not in too advanced a stage, is evidently and greatly improved. Nevertheless, it is necessary to continue the injections week in, week out, or else the state of diabetes rapidly returns. Therefore the question to which an answer is anxiously awaited is whether, after a very long series of Insulin doses, a patient may possibly slowly recover his own power of sugar assimilation. After more cases have been observed we shall probably know, but at present it looks as though the Insulin injc tions would have to be given indefinitely to the patient to prevent a recurrence of the disease.

### THE IRREPRESSIBLE RAT.

ONCE upon a time there was a young " littery gent "—he has since become elderly and distinguished—who, as sometimes happens in a world in which even young literary lions are not perfect, was a trifle " superior," possibly even supercilious. He wrote for a newspaper a biography of a person whom he was graciously pleased to admire, and concluded by remarking that he continued his valuable labours " faint, yet pursuing, to the end." The printer's reader, oddly enough (for printer's readers are mankind's nearest approach to omniscience) did not know the quotation, and placed a query against it on the proof. The contributor impatiently scored out the evidence of ignorance ; but the proof-reader insisted, and repeated his annoying request. Irritated at having to deal with so persistent an ignoramus, that youthful genius hastily scored the derisive word " Rats ! " on the margin of the proof, and when the article appeared it contained the startling statement that its revered subject continued " faint, yet pursuing rats to the end." That, apparently, is what the health authorities of this country will continue to do. Before the war, the damage by rats to the crops cost more than old-age pensions, yet, despite that there is in existence an Act for the Destruction of Rats and Mice, it appears to be almost a dead letter. There have recently been cases of bubonic plague in Paris, and the black rat, which harbours the plague flea, is increasing in numbers in this country. But, the Paris menace apart, it is the height of folly to slacken our destructive efforts. There is nothing whatever to be said for the rat—unlike the charming, elusive mouse, it is not even good-looking. Moreover, it is formidably whiskered, and whiskers are out of fashion. Thus health, appearance, and fashion are all against him. Whether he calls himself *Rattus* or *Norvegicus*, he is equally undesirable—he has even become too numerous for the farmer's wife to continue her beneficent work of cutting off his tail with the carving knife, and being rewarded by a grateful country with a penny a tail.

### BABIES' CRADLE STRAPS.

TWO recent and singularly tragic fatalities in Denmark have drawn attention to the danger of leaving babies in cradles secured by straps. Some arrangement with straps may, indeed, sometimes be necessary when a restless infant has perforce to be left alone, but some straps are much more dangerous than others, and this is one of the lessons of these two accidents in Denmark. There the most usual system of straps consists of four pieces of calico or canvas, 3.3 cm. wide. The first is a belt going round the trunk, and from this belt straps pass over the shoulders, joining the belt on the other side. The fourth strap is usually about 57 cm. long, and a loop at one end runs freely on the belt, while the other end is secured to the cradle. Provided these straps are not too long and are made of inelastic material, there is little risk of a serious accident, but when one of the shoulder straps is missing—and this was the case in one of the accidents referred to—the harness is apt partly to slip off and entangle the infant. Both the children who were strangled were about a year and nine months old—an age when great restlessness is uncompensated by sufficient mental development to help the baby out of its many scrapes. In one case the post-mortem examination showed two narrow grooves running across the front of the neck just under the lower jaw. The publication of these two fatalities has probably served a useful purpose in directing attention to the danger of straps too long and too loosely fitting.

### CAN SALVARSAN KILL SYPHILIS?

THIS question was often raised when Ehrlich first introduced "606," and when he still believed that *sterilisatio magna* could be achieved by one or two large doses. At this period Ehrlich's optimistic claims were not weakened by salvarsan fatalities and failures. The wave of depression which followed them is now passing off, and we are in a much better position to judge of the merits of this drug. In spite of its many enemies, it is still undoubtedly gaining ground, and it is now being advocated not only for treatment, but also for prevention. A Swede, Dr. Karl Heden, has recently published an account of twenty persons who were exposed to infection by syphilitic consorts. It was most important that while these consorts were undergoing treatment for their syphilis, and were still in an infectious stage of the disease, the chances of infection should be reduced to a minimum. Accordingly, prophylactic injections of salvarsan were given to the persons thus exposed, and in every case they remained healthy. In another series of cases prophylactic injections of salvarsan were not given, and of the thirty-six persons exposed to infection as many as thirty contracted syphilis from his or her consort. A Dane, Dr. O. Jersild, has made an interesting comparison between his patients treated with mercury alone, before the salvarsan era, and his patients treated with salvarsan plus mercury. Of his 200 patients treated with mercury alone 80 per cent. relapsed during the first two years, developing infectious secondary eruptions. Of his 169 patients treated in the salvarsan era with arsenobenzol plus mercury, only 20 per cent. relapsed during the two following years. These figures are very remarkable. In the pre-salvarsan era there were 80 per cent. relapses and 20 per cent. effective cures, while in the salvarsan era these proportions were reversed.

### A CITY OF HEALTH.

THAT the bold and public-spirited plan for creating at Welwyn, in Hertfordshire, a new own, which should be a real City of Health, has already achieved a large measure of success is unmistakably indicated by the fact that the Public Works Loan Commissioners have advanced £137,000 to help in its development. Welwyn Garden City, Limited, will receive altogether more than a quarter of a million from this source, which, with its own quarter of a million of capital, should enable it to carry to complete success an enterprise conceived in the best interests of the public health. This model little city is a brilliant example of the new art and science of town-planning. It provides facilities not only for the erection of well-built houses, but for factories and other business undertakings, which will evenually make it at once a social and a commercial community. The ideal town of the future, with its schools and churches, shops and cinemas, works and offices, all hygienically controlled, is thus rapidly coming into being. Placed in a picturesque situation, only 21 miles rom London, and surrounded by a belt of agricultural land, it will be something entirely different from a "dormitory." A light and airy factory looking out upon trees and turf is an even more certain means of keeping the doctor away than the traditional apple a day. Welwyn Garden City grows richer every week by the completion of new works, and when it asks the public, as it does in an advertisement which will be found on another page, to take up the remainder of its six per cent. debentures to the extent of £64,000, it offers a thoroughly sound business proposition. This will complete the issue of its £86,000 worth of debentures, to meet which there are surplus assets to the amount of £274,000. Looking at the progress that has been made, that distinguished authority, Sir H. Trustram Eve, is well justified in declaring that the estate, which must use any profits exceeding seven per cent. for the benefit of the town and its inhabitants, "is now an established success."

